# Supplementary material for: Globodera pallida virulence on major potato resistance has a common genetic basis across Western Europe
Source: PLoS Pathog. 2026 May 6;22(5):e1014201. doi: 10.1371/journal.ppat.1014201 (PMC13160440; doi:10.1371/journal.ppat.1014201)
Supplement: S1 Text — (PDF) [file ppat.1014201.s001.pdf]

S1 Text

METHODS

Statistical analysis on the reliability of the AS-qPCR assay

To assess how well the AS-qPCR-based alternative allele frequency (AAF) matched the sequencing-based AAF, we used linear regression models that followed the formula:

$$AAF_{seq} = \beta_1 * AAF_{qPCR} + \beta_0$$

where AAF_{seq} is the AAF determined by sequencing, AAF_{qPCR} is the AAF determined by qPCR, β_1 is the slope of the model and β_0 is the intercept.

To assess the reliability of the qPCR assay, we assumed that the population is in Hardy-Weinberg equilibrium, so we can calculate the standard error (SE) with the formula:

$$SE = \sqrt{\frac{p(1-p)}{2n}}$$

it should be noted that this assumption is probably true for the D383, Rookmaker and unselected AMPOP populations. For the selected populations, this probably leads to an under-estimation of the aa allele-frequency (since this allele is favourably selected). Assuming Hary-Weinberg equilibrium, the effective population size was calculated with the formula:

$$n = 0.5\frac{p(1-p)}{SE^2}$$

in which *p* is the allele frequency and *n* being the number of individuals (Nei, 1978).

The nonlinear least squares model followed the formula:

$$SE = a(\overline{AAF} - 0.5)^2 + c$$

in which $\overline{AAF}$ is the mean *AAF* of a sample as determined by AS-qPCR. The significance of the curvature parameter (*a*) was tested by calculating a one-sided p-value based on the t-statistic (estimate / standard error), using the residual degrees of freedom from the model fit.

AS-qPCR on mixed populations

To assess the *G. pallida* specificity of the AS-qPCR assay, we performed the assay on *G. rostochiensis* DNA. To assess potential overestimation of the AAF in a mixed population of *G. pallida* and *G. rostochiensis*, we used the formula:

$$AAF_{mix} = \frac{Fr_{pal} * 2^{-Cq_{ALTpal}} + Fr_{ros} * 2^{-Cq_{ALTros}}}{Fr_{pal} * \left(2^{-Cq_{ALTpal}} + 2^{-Cq_{REFpal}}\right) + Fr_{ros} * 2^{-Cq_{ALTros}}}$$

in which AAF_{mix} is the AAF in a mixed population, Fr_{pal} is the fraction of *G. pallida* in a population and Fr_{ros} the fraction of *G. rostochiensis*.

RESULTS

AS-qPCR reliability depends on the community composition, AAF and cyst number

Our AS-qPCR assay showed both accuracy and robustness. Next, we aimed at determining its reliability. Our previous AS-qPCR tests used 20 cysts per sample. However, in field samples, cyst numbers are often limited. Since no sample perfectly represents the population in the field, the reliability of the test depends on the number of cysts that can be obtained from a soil sample. To assess the reliability of the assay on low cyst numbers, we calculated the theoretical standard error (SE) based on the AAF and the number of

individuals tested (*n*). Although each egg within a cyst is genetically unique, they are not genetically independent, due to shared parentage. Therefore, we considered each cyst to be a single individual. Under a worst-case scenario, where the AAF is 0.5, the theoretical SE is 0.079 [sqrt(0.5^2/40)]. In practice, however, the SE observed in our AS-qPCR data was lower, ranging from 0.012 to 0.061 (mean = 0.030; **Supplementary figure 6**). This was expected, as not all tests represent worst-case scenarios and because cyst nematode females are polyandrous and can mate with multiple males (Green et al., 1970; Triantaphyllou & Esbenshade, 1990). Therefore, each cyst contains more genetic diversity than the theoretical model assumes, resulting in a larger effective population size in practice. For each sample, we calculated the effective population size based on the observed mean AAF and the SE. This yields an average effective population size of 187 individuals, which is over nine times higher than the assumed population size of 20. To estimate the SE based on the number of cysts, one could use nine times the number of cysts as the number of individuals used (*n*). When the theoretical SE is too high, reducing the SE by 50% requires a fourfold (2^2) increase in cyst numbers. Conversely, halving the number of cysts increases the SE by a factor 1.41 ($\sqrt{2}$).

Based on the formula used for calculating the SE, we hypothesised that the SE would be AAF dependent. Fitting a quadratic model with a fixed vertex at AAF = 0.5 confirmed this hypothesis (p = 0.016; **Supplementary figure 6**), indicating that AAF_{qPCR} estimates are least precise for AAFs around 0.5 and more reliable at high and low AAFs. Ultimately, the minimum cyst number required for a reliable AS-qPCR assay depends on what someone considers an acceptable level of precision. Notably, the standard error increases as fewer cysts are used, reducing the reliability of the allele frequency estimate.

Running the AS-qPCR on mixed populations

To assess the specificity of the assay, we included *G. rostochiensis* samples as a negative control. The primers targeting the reference allele did not show any amplification on *G. rostochiensis* (Cq > 35.0), which is in line with expectations since the reference allele is not present in *G. rostochiensis*. However, *s*ince the primer targeting the alternative allele has just one mismatch on *Gr-pat-1*, the primers targeting the alternative allele did show minor amplification (Cq ~ 31.3). This may cause a slight overestimation of the AAF in mixed populations. Based on our data, this may lead up to an overestimation by a fraction 0.0024 in case of a populations consisting for 50% of *G. pallida* and 50% *G. rostochiensis.* This overestimation increases when *G. rostochiensis* makes up a larger fraction of the population. However, as the maximum potential overestimation is considerably smaller than the observed standard errors of the AS-qPCR, the potential overestimation of the AAF by *G. rostochiensis* can be ignored.
