# Supplementary figures and images for: Globodera pallida virulence on major potato resistance has a common genetic basis across Western Europe
Source: PLoS Pathog. 2026 May 6;22(5):e1014201. doi: 10.1371/journal.ppat.1014201 (PMC13160440; doi:10.1371/journal.ppat.1014201)

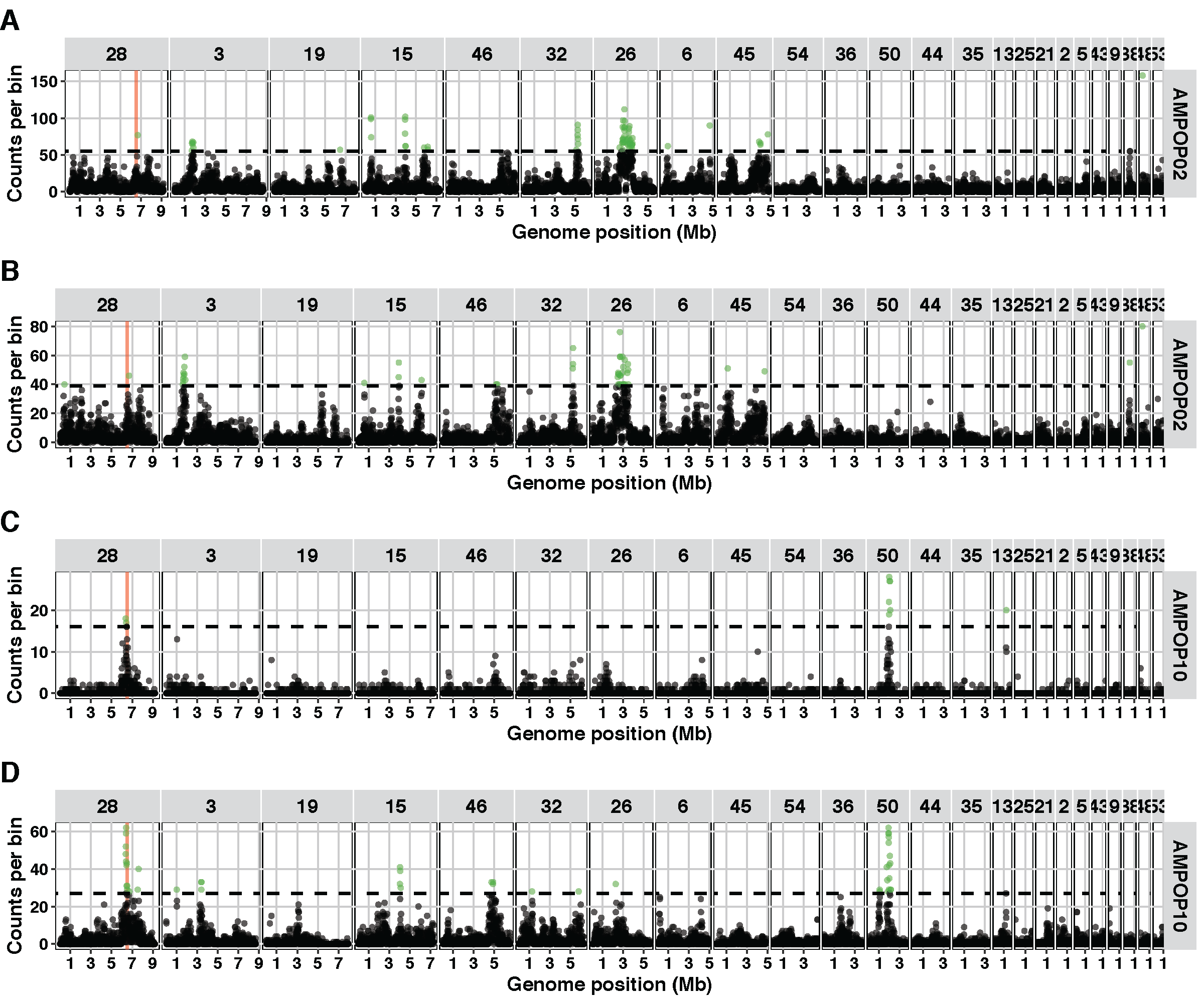

Supplement: S1 Fig — The number of significant SNPs (FDR < 0.05) are plotted per 10kb bin over eight bulk segregant analyses. Bins that are significantly enriched (p < 0.05) are coloured in green. The virulence locus is indicated in red. This was analysed for A and B AMPOP02, C and D AMPOP10. (TIF) [file ppat.1014201.s008.tif]

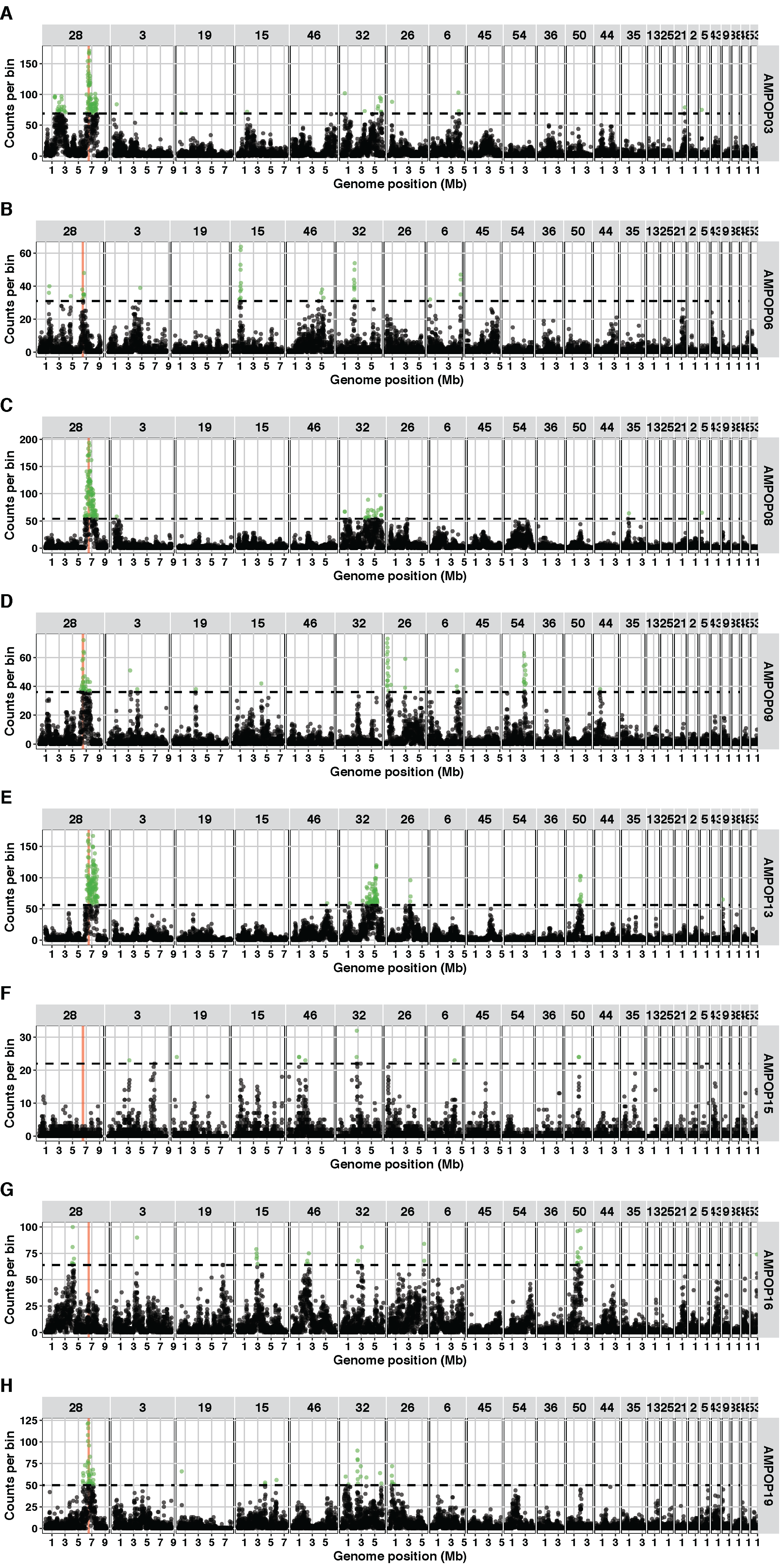

Supplement: S2 Fig — The number of significant SNPs (FDR < 0.05) are plotted per 10kb bin over eight bulk segregant analyses. Bins that are significantly enriched (p < 0.05) are coloured in green. The virulence locus is indicated in red. This was analysed for: A AMPOP03, B AMPOP06, C AMPOP08, D AMPOP09, E AMPOP13, F AMPOP15, G AMPOP16, and H AMPOP19. (TIF) [file ppat.1014201.s009.tif]

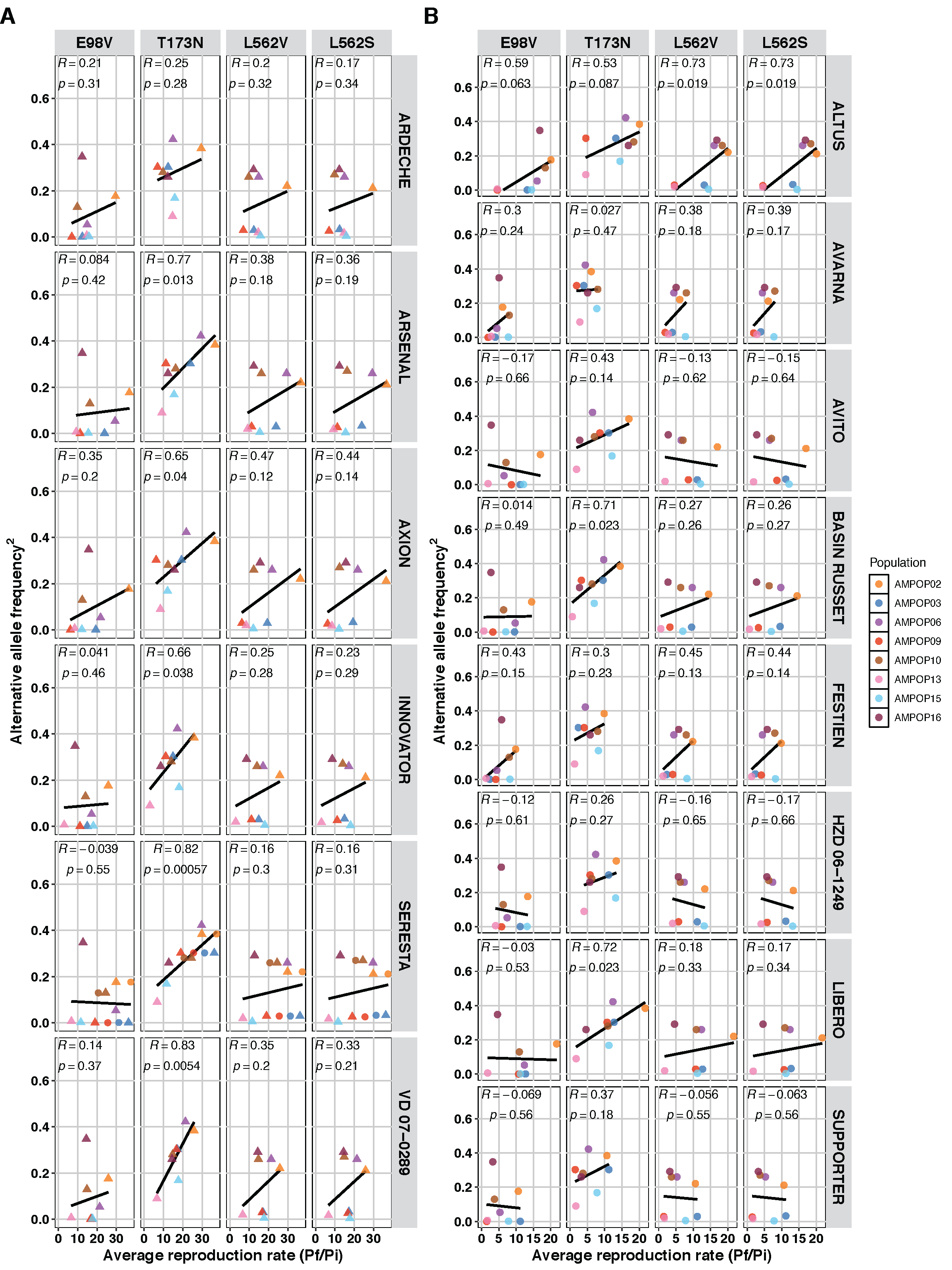

Supplement: S3 Fig — The AAF of T173N significantly correlates with virulence on four of the six ClSER varieties and 2 of the 8 ClFES varieties, indicating that the allele frequency of T173N is a good indication for virulence on GpaVvrn. Statistics are based on linear regression models that are indicated by black lines. (TIF) [file ppat.1014201.s010.tif]

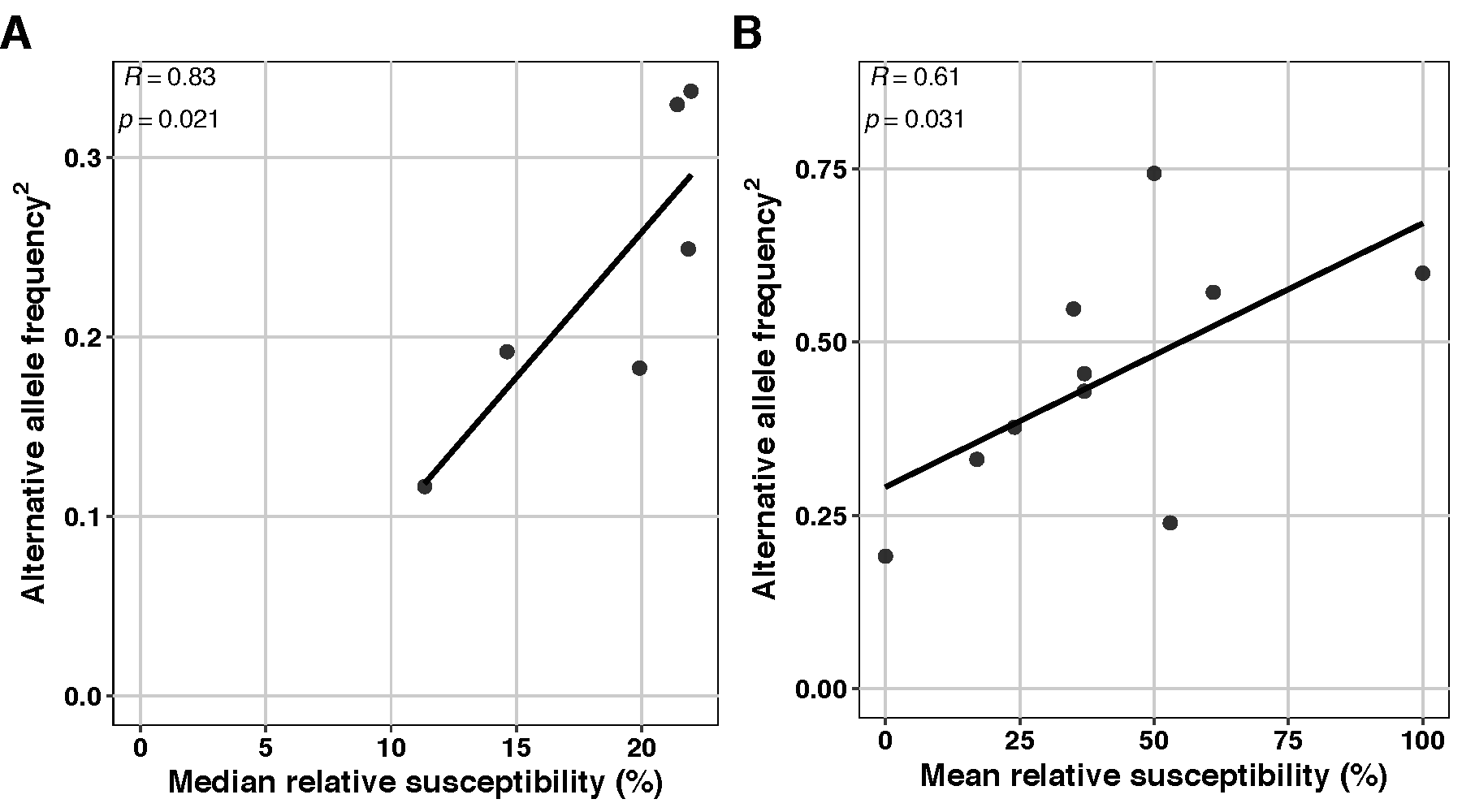

Supplement: S4 Fig — Template DNA is depicted in blue, primers are depicted in magenta, and DNA amplification in green. Created in BioRender (https://BioRender.com/09y6291). B Comparison of two sets of allele-specific qPCR primers: one using LNA-modified primers, with a 3’-terminal locked nucleotide, and one using standard primers. While alternative allele frequency (AAF) estimates from LNA primers significantly correlate with sequencing-based AAFs, they tend to overestimate the AAF in a population, particularly at lower AAFs. In contrast, standard qPCR primers provide a more accurate reflection of the sequencing-based AAF across the tested range. Dashed lines indicate y = x and are added for visual purposes. Statistics are based on linear regression models. (TIF) [file ppat.1014201.s011.tif]

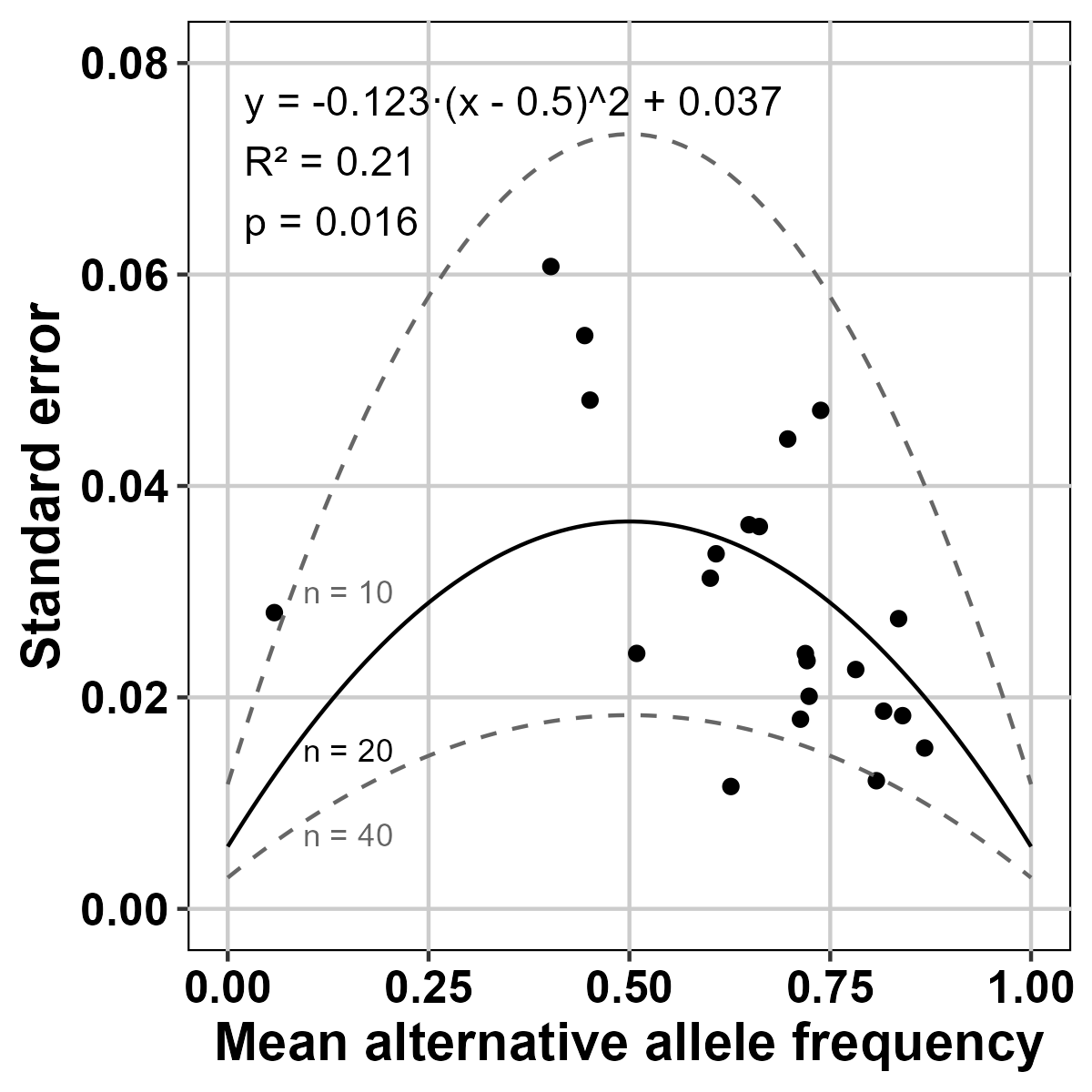

Supplement: S6 Fig — The dashed grey lines indicate the expected standard errors when using halve (n = 10) or double (n = 40) as many cysts in the AS-qPCR. (TIF) [file ppat.1014201.s013.tif]
